# Potassium Alginate Oligosaccharides Alter Gut Microbiota, and Have Potential to Prevent the Development of Hypertension and Heart Failure in Spontaneously Hypertensive Rats

**DOI:** 10.3390/ijms22189823

**Published:** 2021-09-11

**Authors:** Zhen-Lian Han, Meng Chen, Xiao-Dan Fu, Min Yang, Maria Hrmova, Yuan-Hui Zhao, Hai-Jin Mou

**Affiliations:** 1 School of Life Science, Huaiyin Normal University, 111 West Changjiang Road, Huai’an 223300, China; ahanzhenlian@163.com (Z.-L.H.); maria.hrmova@adelaide.edu.au (M.H.); 2 College of Food Science & Engineering, Ocean University of China, 5 Yushan Road, Qingdao 266003, China; chenmeng2838@163.com (M.C.); luna_9303@163.com (X.-D.F.); 3 Yellow Sea Fisheries Research Institute, Chinese Academy of Fishery Sciences, Qingdao 266071, China; minyang89@163.com

**Keywords:** blood pressure, potassium alginate oligosaccharides, gut microbiota, heart failure

## Abstract

Food-derived oligosaccharides show promising therapeutic potential in lowering blood pressure (BP), but the mechanism is poorly understood. Recently, the potential role of gut microbiota (GM) in hypertension has been investigated, but the specific GM signature that may participate in hypertension remains unclear. To test the potassium alginate oligosaccharides (PAO) mechanism in lowering BP and specific microbial signature changes in altering GM, we administered various dosages of PAO in 40 spontaneously hypertensive rats for a duration of six weeks. We analyzed BP, sequenced the 16S ribosomal DNA gene in the cecum content, and gathered RNA-seq data in cardiac tissues. We showed that the oral administration of PAO could significantly decrease systolic BP and mean arterial pressure. Transcriptome analyses demonstrated that the protective effects of developing heart failure were accompanied by down-regulating of the Natriuretic Peptide A gene expression and by decreasing the concentrations of angiotensin II and atrial natriuretic peptide in plasma. In comparison to the Vehicle control, PAO could increase the microbial diversity by altering the composition of GM. PAO could also decrease the ratio of Firmicutes to Bacteroidetes by decreasing the abundance of *Prevotella* and *Phascolarctobacterium* bacteria. The favorable effect of PAO may be added to the positive influence of the abundance of major metabolites produced by Gram-negative bacteria in GM. We suggest that PAO caused changes in GM, and thus, they played an important role in preventing the development of cardiovascular disease.

## 1. Introduction

Hypertension (HTN) has been identified as the leading cause of mortality and disability worldwide because of the high frequency and concomitant risks of cardiovascular diseases (CVD), including stroke and heart failure (HF) [1,2,3]. Stroke, heart disease, and chronic obstructive pulmonary diseases are the leading causes of death in China [4]. Therefore, sustainable methods for reducing high blood pressure (BP) are urgently needed to combat emerging hypertension in China and worldwide [4,5]. In recent years, researchers found that the lowering BP alginate oligosaccharides include sodium alginate oligosaccharides (SAO) [6,7] and potassium alginate oligosaccharides (PAO) [8], but the mechanism of their action remains unknown.

Alginate oligosaccharides are indigestive in the small intestine but fermented in the colon, where they are colonized by commensal bacteria, which are known as gut microbiota (GM) [9]. Accumulated evidence suggested that GM were associated with various diseases, such as colorectal cancer, liver cirrhosis, arthritis, type 2 diabetes, and atherosclerosis [10,11,12,13,14]. Emerging evidence supported that the changes in the intestinal flora could regulate BP [15,16], and gut dysbiosis is an important causal factor for the pathogenesis of HTN [17]. Marques et al. reported that a high-fiber diet could prevent the development of HTN via changing GM in DOCA–Salt hypertensive mice [18]. Hidalgo et al. suggested that an extra virgin olive oil-enriched diet also reduced HTN-related profile changes of GM in spontaneously hypertensive rats (SHR) [19]. Captopril, an angiotensin-converting enzyme inhibitor (ACEi), could influence the brain–gut axis to maintain the antihypertensive effect in SHR [20] but have adverse side effects, such as the reduced renal function and angioedema [21]. Regulation of intestinal microbiota through diet interventions may be a new strategy for the nutritional treatment of HTN [22].

Studies showed that oligosaccharides could also have beneficial activities to human/animal health through interaction with gut microbiota [23]. Alginate oligosaccharides improve lipid metabolism and inflammation by modulating *Akkermansia muciniphila*, *Lactobacillus reuteri*, and *Lactobacillus gasseri* in high-fat diet fed mice and increase the “beneficial” microbes such as Leuconostocaceae in mice with small intestinal mucositis [24,25]. In our previous study based on our data of the 16S ribosomal DNA (rDNA) gene sequencing, we found out that alginate oligosaccharides altered the microbiota composition and decreased the ratio of Firmicutes and Bacteroidetes in pig fecal bacteria [26]. Therefore, the specific microbial signature associated with HTN and the mechanism lowering BP need to be revealed after interventions with alginate oligosaccharides.

To address the questions specified above, we performed 16S rDNA gene sequencing of cecum samples from 30 SHR of Vehicle, PAO, KCl, and Captopril-treated diets. The cardiac RNA-seq data were also analyzed and pointed at the crucial differential gene expression of associated genes to reveal the mechanism of PAO in lowering BP.

## 2. Results

### 2.1. HPAO and LPAO Decreased BP in SHR

**Figure 1:**
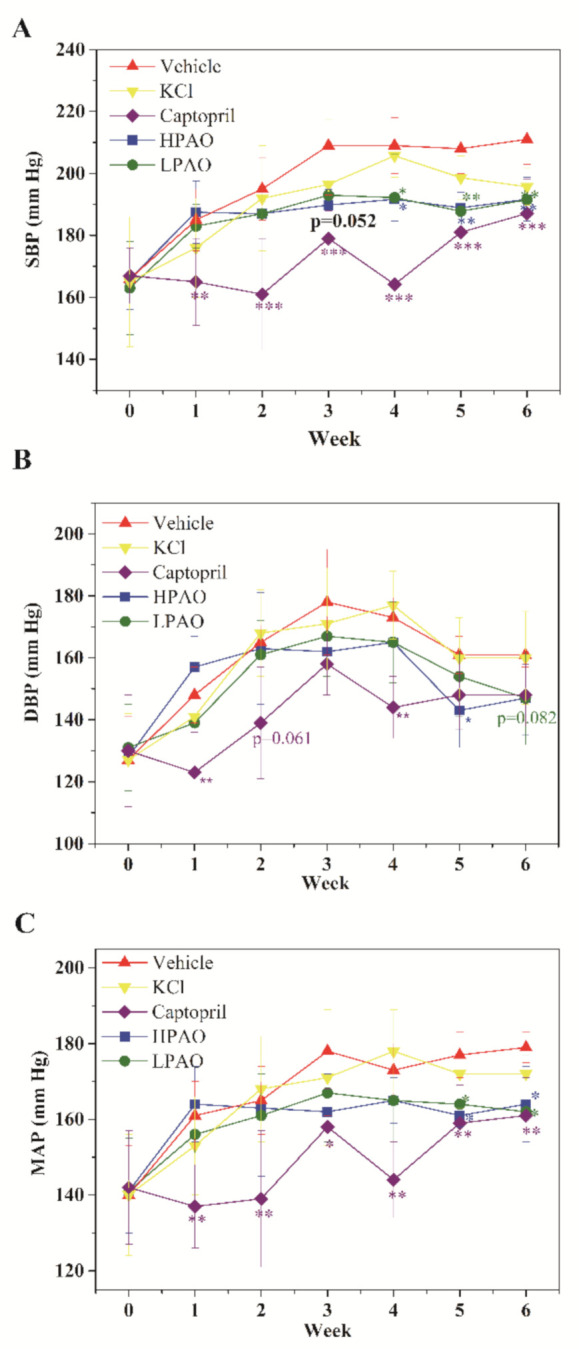
Vehicle, KCl, Captopril, HPAO, and LPAO modulate (**A**) SBP, (**B**) DBP, and (**C**) MAP in the SHR model. Values are mean ± SD (*n* = 8). * *p* < 0.05, ** *p* < 0.01, *** *p* < 0.001 vs. Vehicle.

The SBP, DBP, and MAP parameters were measured to assess the effect on BP during the 6-week period (Figure 1). In Vehicle, SBP increased along within three weeks’ administration. From week 4 to 6, SBP was stable, and a similar trend was observed for MAP, while DBP slowly decreased. After 6-week administration, SBP increased by more than 40 mmHg in total. This agreed with the previous study of SHR [27].

In the present study, HPAO decreased SBP significantly compared to Vehicle on week 3 (mean ± SD 189.8 ± 5 vs. 209 ± 16, *p* = 0.052, Figure 1A). Furthermore, Figure 1A showed that the administration of HPAO significantly decreased SBP from week 4 to week 6 versus the Vehicle-treated group (mean ± SD 191.7 ± 7 mmHg vs. 209 ± 9 mmHg, 188.9 ± 5 mmHg vs. 208 ± 8 mmHg, 191.7 ± 7 mmHg vs. 211 ± 8 mmHg; *p* < 0.05, *p* < 0.01 and *p* < 0.01, respectively, Figure 1A). MAP was mirrored by the similar decreases on weeks 5 and 6 (Figure 1C). These data showed that HPAO vs. Vehicle decreased SBP and MAP. The oral administration of LPAO at a dose of 300 mg/kg also decreased SBP from week 4 to week 6 (192.2 ± 14 mmHg, 187.8 ± 12 mmHg, 191.5 ± 17 mmHg, *p* < 0.05, *p* < 0.01 and *p* < 0.01, respectively; Figure 1). Similar decreases were found in levels of MAP with LPAO on weeks 5 and 6 (Figure 1C). To elucidate the potential effect of potassium, we treated SHRs at the dose of 114 mg/kg KCl. We found that 114 mg/kg KCl did not significantly change BP in the SHR model, suggesting that phenotypic differences may have been caused by the alginate oligosaccharides in SHR. Captopril significantly reduced SBP and MAP compared to the Vehicle-treated group from week 1 to week 6, and DBP decreased relatively weakly similar to HPAO and LPAO (Figure 1A–C).

### 2.2. HPAO Interventions Attenuated Cardiac Fibrosis

**Figure 2:**
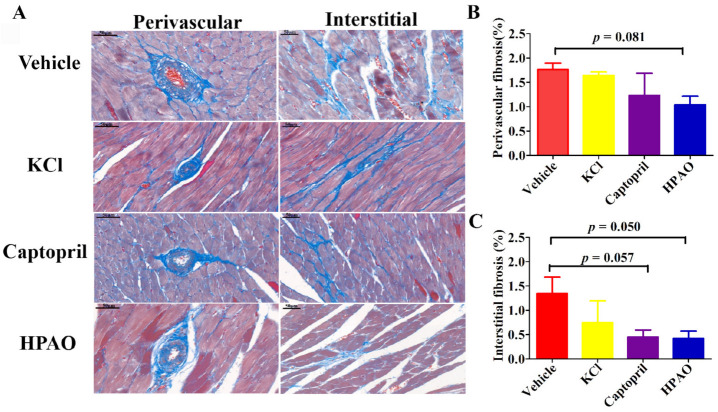
HPAO reduces cardiac fibrosis in SHR. (**A**) Representative histological images of obtained via Masson trichrome staining for each treatment (scale bar = 50 μm). Compared to Vehicle, HPAO caused a mild decrease in (**B**) cardiac perivascular fibrosis, while (**C**) interstitial fibrosis was significantly reduced by HPAO. Values are mean ± SEM. One-way ANOVA with Least Significant Difference (LSD) adjustment for multiple comparison was used.

Perivascular and interstitial cardiac fibrosis were observed in SHR after six weeks (Figure 2). The perivascular volume fraction decreased from 1.76 ± 0.13% in the Vehicle-treated group to 1.04 ± 0.17% in the HPAO group (*p* = 0.081). The interstitial volume fraction also decreased in SHR from the values of 1.34 ± 0.34% in the Vehicle-treated group to 0.42 ± 0.15% in the HPAO group (*p* = 0.050). Again, Captopril (0.45 ± 0.14%, *p* = 0.057) attenuated the perivascular and interstitial volume fraction but could not be significantly compared to the Vehicle-treated group (Figure 2B,C). These data indicated that the 6-week administration of HPAO could attenuate interstitial cardiac fibrosis, which could be an important therapeutic target for HF [28].

### 2.3. Cardiac Transcriptome

**Figure 3:**
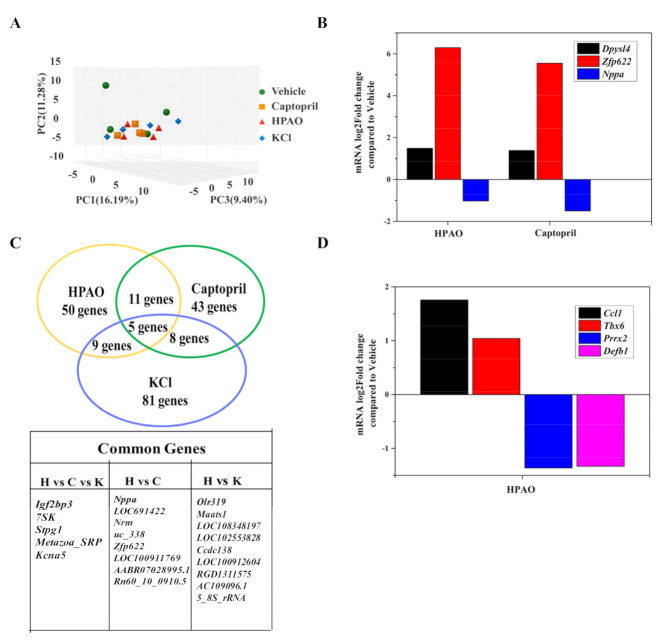
Cardiac transcriptome of SHR fed with HPAO, Captopril, KCl, and Vehicle diets. (**A**) PCA analysis, (**B**) common genes between HPAO, Captopril, and KCl compared to the Vehicle group, (**C**) differentially expressed common genes between the HPAO and Captopril groups, and (**D**) differentially expressed genes in the HPAO group.

To support the underlying mechanism, transcriptome profiling of cardiac samples from the HPAO, Captopril, KCl, and Vehicle-treated groups was carried out. As shown in Figure 3A–D, Principal Component Analysis (PCA) showed that the HPAO, KCl, and Captopril-treated groups differed mildly compared to the Vehicle-treated group (Figure 3A). In the heart, 32,883 genes were examined in total. Of these, 563 genes were differentially expressed in the HPAO-treated group (*p*-value < 0.05, Table S1), while 626 genes were differentially expressed in the Captopril-treated group (Table S2) compared to the Vehicle-treated group. Sixteen genes were shared between the HPAO and Captopril-treated groups (Figure 3C), whereby the *Nppa* gene was down-regulated; the expression of the *Nppa* gene is mostly associated with hypertrophic ventricle [29], HTN [30], and HF [31]. On the other hand, the up-regulated genes thought to have a role in albuminuria [32] and cardiac cell death [33] were Zinc finger protein 622 (*Zfp622*) and Dihydropyrimidinase like 4 (*Dpysl4*), respectively (Figure 3B). Additionally, four other genes were markedly differentially expressed between the Vehicle-treated and HPAO groups (Figure 3D); these genes are the Paired mesoderm homeobox protein 2 (*Prrx2*) and the T-box transcription factor 6 (*Tbx6*) that are associated with cell migration [34] and cardiomyocyte regeneration, respectively [35]. On the other hand, Beta-defensin 1 (*Defb1*) and the C-C motif chemokine ligand 1 (*Ccl1*) are related to HTN.

Differential expressed genes (DEGs) between the HPAO and Vehicle groups were further analyzed using the Gene Ontology (GO) bioinformatics resource, whereby cellular components and biological process involved in DEGs in the Vehicle vs. HPAO-treated groups were analyzed (Tables S3 and S4). The molecular functions of the DEG entries were classified into ten categories, including binding, catalytic activity, transporter activity, nucleic acid binding transcription factor activity, signal transducer activity, and molecular transducer activity (Figure S1A). Among them, DEG entries related to binding, catalytic activity, and transporter activities accounted for 49%, 23%, and 8%, respectively. Forty-eight KEGG pathways enriched by DEG entries between the HPAO and Vehicle-treated groups are summarized (Table S5). As expected, seven significantly enriched disease hallmark pathways that were identified (Figure S1B), including MAPK, mTOR, and the chemokine signaling pathway, are associated with HTN-induced renal damage [36], cardiac hypertrophy [37], and vascular remodeling [38], respectively. Shigellosis and Legionellosis were also significantly enriched by HPAO.

**Figure 4:**
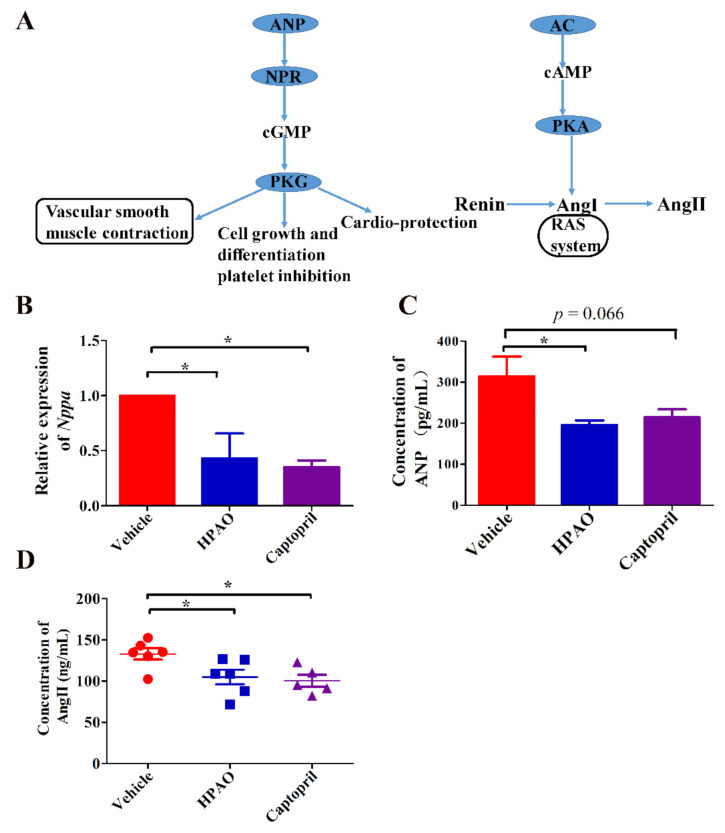
The influence of changes in *Nppa* expression level on the pathway. (**A**) Summarized pathways related to cardia, where *Nppa* is involved, (**B**) mRNA levels of *Nppa* expression, concentrations of (**C**) ANP and (**D**) AngII. Data were mean ± SEM (*Nppa*, ANP, *n* = 3). Significance was accepted at * *p* < 0.05. One-way ANOVA with LSD adjustments for multiple comparison was used.

The *Nppa* gene participated in ten pathways. Five out of the 10 signaling pathways were up-regulated by Captopril, such as aldosterone synthesis and secretion and the cAMP, thermogenesis, and oxytocin signaling pathways (Figure S2). The oxytocin signaling pathway was also up-regulated in the HPAO group (FDR *q*-value = 0.78). To understand which pathway was specifically affected by the *Nppa* gene, known regulators of associated pathways are summarized (Figure 4A). To verify the hub of the *Nppa* gene expression, the mRNA expression level of *Nppa* (Figure 4B) and the ANP protein level (Figure 4C) were analyzed. To verify which of the downstream regulators in these pathways change, the plasma level of AngII was examined (Figure 4D). These data suggested that HPAO could down-regulate the mRNA level of *Nppa* and reduce the synthesis of ANP and AngII at the protein levels in the plasma, as these levels differed significantly (Figure 4C,D, *p* < 0.05) between the HPAO and Vehicle-treated groups.

### 2.4. PAO Altered Gut Microbiota Dysbiosis in SHR

**Figure 5:**
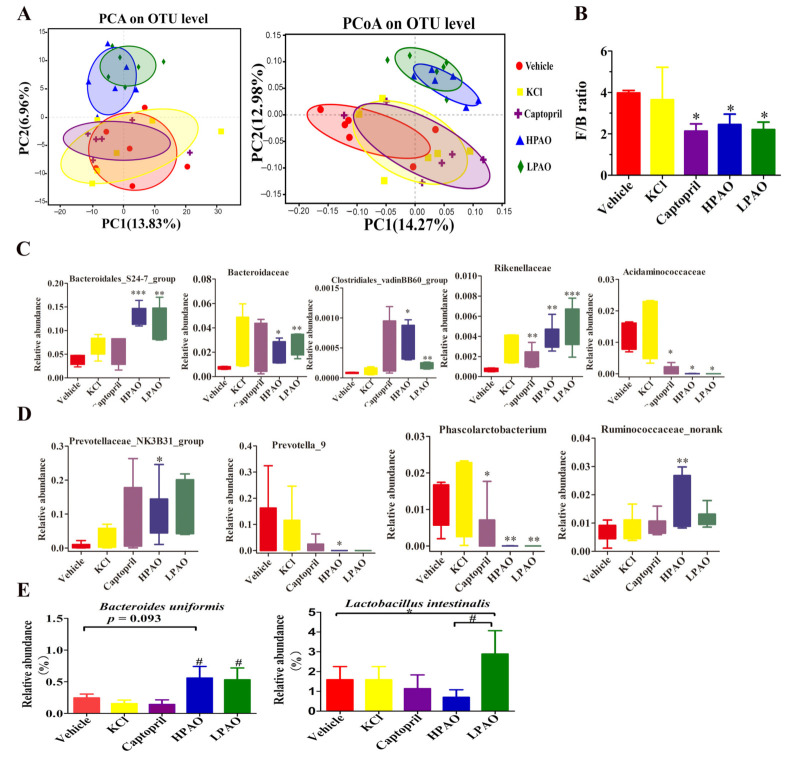
The effect of Vehicle, KCl, Captopril, HPAO, and LPAO supplementation in GM. (**A**) HPAO and LPAO alter the composition of GM significantly compared to the KCl, Captopril, and vehicle groups; the data indicate unweighted UniFrac PCA and unweighted UniFrac PCoA. (**B**) The Firmicutes to Bacteroidetes ratio, as a marker of gut dysbiosis, is significantly lower in HPAO, LPAO, and Captopril (*p* < 0.05). One-way ANOVA with Tukey’s adjustments for multiple comparison was used. (**C**) The relative abundance of significant families in the top 24 entries between the Vehicle, KCl, Captopril, HPAO, and LPAO groups. Statistical analysis was performed using *t*-test. (**D**) Relative abundance of significant genera in top 40 between the Vehicle, KCl, Captopril, HPAO, and LPAO groups. One-way ANOVA with LSD adjustments for multiple comparison was used. (**E**) PAO had higher levels of bacteria from the *Bacteroides uniformis* and *Lactobacillus intestinalis* species than the Vehicle group (*p* = 0.093). Data were mean ± SEM * *p* < 0.05, ** *p* < 0.01, *** *p* < 0.001 vs. Vehicle, ^#^ *p* < 0.05 vs. KCl and Captopril groups, respectively. One-way ANOVA with LSD adjustments for multiple comparison was used.

To investigate if PAO alters the GM structure and composition in SHR, we sequenced the 16S rDNA gene in the cecum content. Through alpha diversity analyses, Shannon, Simpson, Ace, and Coverage indices were compared between PAO, KCl, and Captopril with Vehicle-treated groups. The Ace Index was significantly increased (*p* = 0.03) between the HPAO and Vehicle groups (Table S6 and Figure S3). The Shannon Index was observed to be significantly different (*p* = 0.076) between the KCl- and Vehicle-treated groups. The data of the β diversity analysis, PCA, and the Principal Coordinates Analysis (PCoA) showed that HPAO and LPAO both altered the composition of GM significantly compared to the KCl, Captopril, and Vehicle diets (Figure 5A). This was supported by the differences in the relative abundance in bacterial phyla (Figure S4A), families (Figure S4B), and at genera levels (Figure S4C) between the groups. At the phylum level, the dominant bacteria in SHR were Firmicutes and Bacteroidetes (Figure S4A), representing up to 95% of the total amount of bacteria. The ratio of Firmicutes and Bacteroidetes, as a marker of gut dysbiosis, was five-fold higher in SHR compared to that in Wistar–Kyoto (WKY) rats [14]; this ratio was significantly lowered to nearly one-half in the HPAO, LPAO, and Captopril group compared to the Vehicle-treated group (*p* < 0.05) (Figure 5B). At the family level, the dominant bacteria (relative abundance > 0.01) were Lachnospiraceae, Ruminococcaceae, Prevotellaceae, Bacteroidales_S24-7_group, Lactobacillaceae, Bacteroidaceae, Christensenellaceae, Acidaminococcaceae, and Verrucomicrobiaceae (Figure S4B). In the top 24 families, the relative abundance of Bacteroidales_S24-7_group, Rikenellaceae, and Bacteroidaceae that belong to Bacteroidetes was significantly higher in the PAO group compared to the Vehicle-treated group, while the relative abundance of Acidaminococcaceae (classified in Firmicutes) was significantly lower both in the HPAO and Captopril groups (*p* < 0.05) (Figure 5C). The relative abundance of the Clostridiales_vadinBB60_group that belongs to Firmicutes was significantly higher in the PAO groups compared to the Vehicle-treated group, but the relative abundance of the Clostridiales_vadinBB60_group was less than 0.0010. Therefore, the contribution of the Clostridiales_vadinBB60_group to the relative of abundance of Firmicutes was small. At the top 40 genus level, the relative abundance of *Prevotella_9* (0–0.3) and *Phascolarctobacterium* (<0.05) was significantly decreased in the HPAO group compared to the Vehicle-treated group (*p* < 0.05, *p* = 0.005, respectively), while *Prevotellaceae_NK3B31_group* and *Ruminococcaceae_norank* were increased significantly (*p* < 0.05, *p* = 0.000, respectively, Figure 5D). At the species level, the relative abundance of *Bacteroides uniformis* was high in the HPAO and LPAO groups, but this bacterium had low levels in the KCl, Captopril, and Vehicle groups (*p* = 0.093, Figure 5E). The abundance of *Lactobacillus intestinalis* was significantly higher in the LPAO group compared to the Vehicle-treated group (*p* = 0.038, Figure 5E). Furthermore, changes in bacteria between the PAO, Captopril, and Vehicle groups were analyzed by Linear discriminant analysis effect size (LEfSe) (Figure S5 and Table S7). The abundance of Coriobacteriaceae was reported to show a significant increase in hypertensive rats fed on the high-fat diet [39]; the abundance was higher in the Vehicle-treated group compared to those in the HPAO, LPAO, and Captopril groups. These data indicated that PAO could alter the relative abundance of Firmicutes and Bacteroidetes to modify gut dysbiosis, which was supported by changes of the relative abundance at the family, genus, and species levels.

### 2.5. 16S rDNA Gene Function Analysis

**Figure 6:**
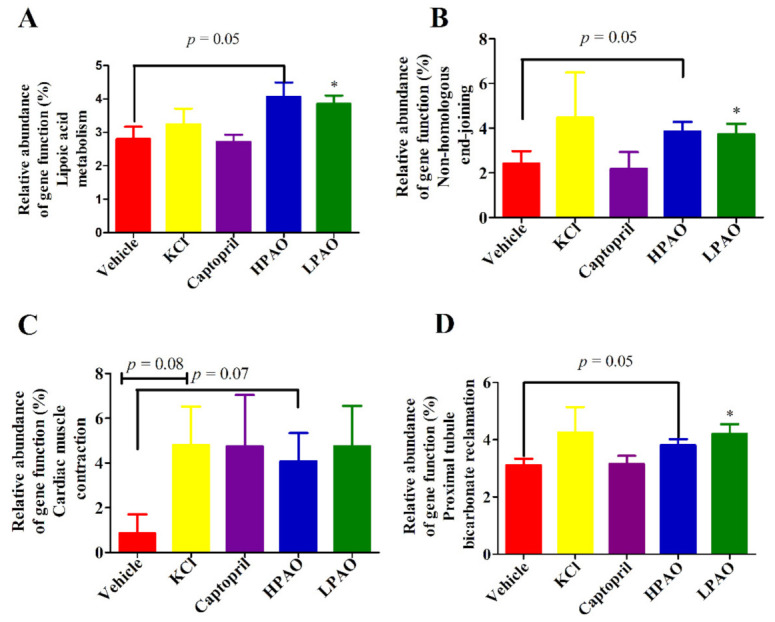
Selected biological pathways and functional categories of GM displaying differences in the relative abundance in GM. Relative abundance of the gene function (%). (**A**) Lipoic acid metabolism, (**B**) Non-homologous end-joining, (**C**) Cardiac muscle contraction, and (**D**) Proximal tubule bicarbonate reclamation. Data were mean ± SEM. Statistical analysis was performed using *t*-test * *p* < 0.05 vs. Vehicle.

To understand how they operate, 16S rDNA gene sequence data were further analyzed by PICRUSt analysis to predict the function of PAO. In total, 196 KEGG pathways and 25 Clusters of Orthologous Groups (COG) functional categories were predicted from the Operational Taxonomic Unit (OTU) dataset. We observed that four KEGG pathways were particularly affected by the PAO (Figure 6). Lipoic acid metabolism, non-homologous end-joining, and proximal tubule bicarbonate reclamation were significantly enhanced by LPAO (*p* < 0.05, Figure 6A,C,D). It has been previously reported that lipoic acid, an antioxidant, could lower BP in SHR [40] and improve the endothelial function in metabolic syndrome patients, which is associated with increased Ang II activity [41]. These data illustrated that an increase in the relative abundance of the 16S rDNA gene may impact the lipoic acid metabolism, which is evidence that PAO could lower BP.

## 3. Discussion

In the present study, we investigated if HPAO could lower BP and alter the GM structure and composition to be able to prevent the cardiac effects of CVD. As expected, we found that HPAO could lower BP in the SHR model. These findings were similar to those obtained with SAO and PAO, whereas these preparations could attenuate BP in a genetic rat model of human HTN and deoxycorticosterone acetate (DOCA) salt-induced HTN, respectively [6,8]. Furthermore, we found that HPAO was able to reduce cardiac fibrosis, while we did not observe any significant changes in the KCl group. A previous study [42] indicated that alginate oligosaccharides could alleviate the myocardial reperfusion injury, in part by inhibiting oxidative stress and endoplasmic reticulum stress-mediated apoptosis. However, in this study, we also observed an alternative molecular mechanism by analyzing the data of the cardiac transcriptome in the HPAO, Captopril, and Vehicle-treated groups. The *Nppa* gene, encoding for the ANP, is involved in regulation of the cAMP signaling pathway and renin–angiotensin system (RAS), while cAMP signaling could ameliorate HTN [43], where the cardiac myocyte cAMP level and the myocardial contractile function are regulated by the phosphodiesterase 3A [44]. Ang II could influence the BP level through regulating the vascular contraction and relaxation [45]. ANP is supposed to be beneficial in heart failure and hypertension [46]. However, in this study, the concentration of Ang II and ANP were decreased compared to the Vehicle group in SHR. Soualmia H et al. [47] reported that AngII can affect the changes of ANP by the NO in SD rats. It is known that the concentration of AngII is a key mediator in hypertension [48]. Therefore, as HPAO could lower BP and prevent the development of HF in the SHR model, we suggested that HPAO may regulate the expression of *Nppa* and then regulate the cAMP signaling pathway and RAS.

**Figure 7:**
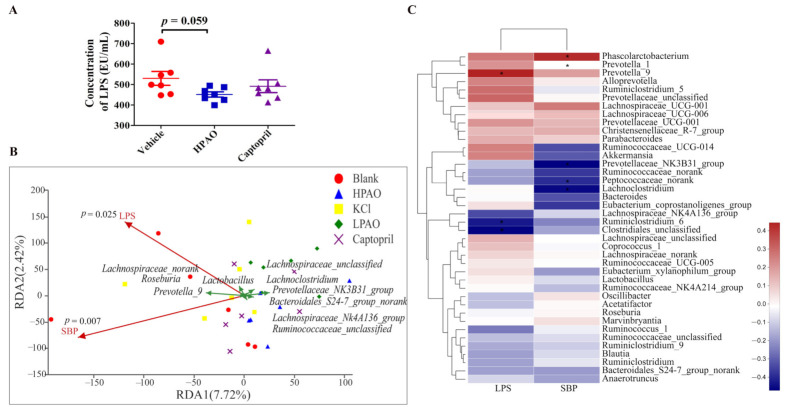
Correlation analysis between changes of plasma LPS level, intestinal bacteria, and SBP. (**A**) The concentration of LPS in Vehicle, HPAO, and Captopril. Data were mean ± SEM (*t*-test, *n* = 7). (**B**) The redundancy analysis between LPS, SBP, and ten taxa. (**C**) The correlation analysis between LPS, BP, and GM in the top twenty entries at the family and genera levels (* *p* ≤ 0.05).

HPAO could also significantly enriched the Shigellosis and Legionellosis signaling pathways (Figure S1). LPS, a component of the outer membrane of the Gram-negative bacteria, is involved in the Legionellosis signaling pathway. For this reason, we determined plasma LPS. The concentration of plasma LPS was decreased by HPAO after a six-week intervention compared to Vehicle (Figure 7A) and significantly influenced the populations of bacteria in the top ten genus distribution (*p* = 0.025) (Figure 7B). Notably, as observed in the present study, the relative abundance of the Gram-negative bacteria, such as *Phascolarctobacterium* and *Prevotella_9* decreased nearly to zero (Figure 5D), and this was positively correlated with LPS and SBP, respectively (Figure 7C) after HPAO intervention. Higher relative abundances of Gram-negative bacteria including *Phascolarctobacterium* and *Prevotella* were related to the higher BP in HTN patients [17,49]. In the hypertensive state, more gut permeability was present and LPS translocated from the gut to the circulation and induced systemic inflammation, which is linked to hypertension [50,51]. In addition, LPS is also known as the mechanistic biomarker of CVD [52]. Our finding demonstrated that HPAO decreased BP, which is associated with modifying gut dysbiosis, lowering the abundance of *Prevotella_9* and *Phascolarctobacterium*, and reducing LPS metabolites. We suggested that further studies are needed to verify if the oral administration of HPAO could lower BP by modulation of the abundance of *Prevotella* and *Phascolarctobacterium*.

To understand the role of another metabolite of GM, cecum short-chain fatty acids (SCFAs) were measured (Figure S6). As for SCFAs changes, compared to Vehicle, no significant differences were observed in acetic acid, propionic acid, and butyric acid levels in the HPAO group, which is consistent with the results of a previous report in WKY and SHR [27]. These analyses also provided evidence that the changes of the GM composition correlated with the development of hypertension. In the present study, compared to Vehicle, the levels of propionic acid-producing bacteria, *Bacteroides uniformis*, did not differ significantly in the HPAO group (Figure 5E). However, we could not rule out that SBP changes could correlate with the concentration of SCFAs. Several studies reported that acetate and propionate levels could attenuated cardiac fibrosis [18,53]. Notably, the level of the lactic acid level was raised after six weeks in the HPAO group after administration. Levels of *Lactobacillus intestinalis* were higher in the LPAO group compared to Vehicle. Candesartan may attenuate chronic low-grade inflammation by counteracting decreased levels of lactic acid-producing genus *Lactobacillus* [54]. Levels of SCFA-producing microbes were negatively correlated with fecal SCFA levels [53], and we also found the higher concentration of lactic acid and the lower abundance of *Lactobacillus* in the HPAO group. In the future, it will be necessary to undertake intervention studies with oral SCFA that could help to elucidate the pathways or effects of these metabolites on BP [55].

It is also important to point out that HPAO decreased BP by regulating the ratio of Firmicutes to Bacteroidetes, lowering the relative abundance of *Prevetolla_9* and *Phascolarctobacterium*, and reducing the plasma LPS level. Furthermore, HPAO attenuated cardiac fibrosis, possibly by regulating the expression of *Nppa*, the cAMP signaling pathway, and RAS to prevent the development of CVD.

## 4. Materials and Methods

### 4.1. Preparation of PAO

PAO was prepared by the enzymatic hydrolysis of potassium alginate (Qingdao Hyzlin Biology Development Co. Ltd., Qingdao, China) method as described previously with slight modifications [26]. In brief, 5% (*w*/*v*) potassium alginate was mixed with the alginate lyase (28 U/mL) and incubated for 6 h at 37 °C to prepare PAO. After hydrolysis, the sample was centrifuged (23,275× *g*, 20 °C, 15 min), and the final product was obtained through a three-fold precipitation of the supernatant by 95% (*v*/*v*) ethanol. The chemical composition and the molecular mass of the hydrolysate (Table S8) were determined using the previously described method [26].

### 4.2. Animal Model and Treatment

The experimental protocol was approved by the Committee on the Ethics of Animal Experiments of Ocean University of China (Approved protocol ID SCKK 2016–0011). SHR (body weight varied between 175 and 215 g, Male, 8 weeks old) were obtained from Vital River Laboratory Technology Co. Ltd. (Beijing, China). During experiments, rats were housed in a room maintained under the 12 h light/dark cycle at 22 °C. Rats had free access to fresh water and were fed the Maintenance Purified Diet (GB 14924.3-2010) ad libitum throughout experiments. Rats were randomly assigned to five groups (*n* = 8): Vehicle-treated group (Vehicle), 600 mg/kg PAO-treated group (HPAO), 300 mg/kg PAO-treated group (LPAO), 114 mg/kg KCl-treated group (KCl), and 10 mg/kg Captopril-treated group (Captopril) that were refreshed twice per week. Vehicle (volume-matched distilled water), HPAO, LPAO, KCl, and Captopril were administered orally once daily for six weeks. Systolic blood pressure (SBP), diastolic blood pressure (DBP), and mean arterial pressure (MAP) were measured using the tail-cuff method on the rat blood pressure monitoring system (BP-2010A System, Softron, Beijing, China) every week. After six weeks of treatment, all rats were anaesthetized and sacrificed by ether. Blood samples were collected and centrifuged (1000× *g*, 20 min, 4 °C) to isolate blood cells. Rats were weighed and their SBP was measured every week; the body weight changes are shown in Figure S7. Concentrations of plasma lipopolysaccharide (LPS), angiotensin II (Ang II), and the atrial natriuretic peptide (ANP) were determined using the commercial ELISA Kits (Shanghai Elisa Biotech Co., Ltd., Shanghai, China) according to the manufacturer’s protocols.

### 4.3. Gut Microbiome

The cecum contents were collected rapidly and stored at −80 °C. Microbial DNA was extracted from six samples per group using the E.Z.N.A.^®^ soil DNA Kits (Omega Bio-tek, Norcross, GA, USA) according to the manufacturer’s protocols. DNA concentration and purity were determined by NanoDrop 2000 (Thermo fisher Scientific Inc., Wilmington, NC, USA), and DNA quality was checked on 1% (*w/v*) agarose gels. The V3–V4 hypervariable regions of the bacteria 16S rDNA gene were amplified using the forward 5′-ACTCCTACGGGAGGCAGCAG-3′ (338F) and reverse 5′-GGACTACHVGGGTWTCT AAT-3′ (806R) primers in the thermocycler PCR system (GeneAmp 9700, American Butter Institute, Arlington, VA, USA). PCR reactions were amplified after 3 min of denaturation at 95 °C, 27 cycles of 30 s at 95 °C, 30 s for annealing at 55 °C, 45 s for elongation at 72 °C, and the final extension at 72 °C for 10 min. PCR reactions were performed in triplicate 20 μL mixture containing 4 μL of 5× FastPfu Buffer (pH 9.2), 2 μL of 2.5 mM dNTPs, 0.8 μL of each primer (5 μM), 0.4 μL of the FastPfu Polymerase, and 10 ng of template DNA. PCR products were extracted from 2% (*w/v*) agarose gels, purified using the AxyPrep DNA Gel Extraction Kit (Axygen Biosciences, Union City, CA, USA), and quantified using QuantiFluor™-ST (Promega, Madison, WI, USA) according to the manufacturer’s protocol.

Purified amplicons were pooled in equmolar quantities and paired-end sequenced (2 × 300) on an Illumina MiSeq platform (Illumina, San Diego, CA, USA) according to protocols established by the Shanghai Majorbio Bio-Pharm Technology Co. Ltd. (Shanghai, China).

The raw 16S rDNA gene sequencing reads were demultiplexed, quality filtered by fastp version 0.20.0, and merged by FLASH version 1.2.7 with the following criteria: (i) the 300 bp reads were truncated at any site receiving an average quality score of <20 over a 50 bp sliding window, the truncated reads shorter than 50 bp were discarded, and reads containing ambiguous characters were also discarded; (ii) only overlapping sequences longer than 10 bp were assembled according to their overlapped sequence. The maximum mismatch ratio of overlap region is 0.2. Reads that could not be assembled were discarded; (iii) samples were distinguished according to the barcode and primers, and the sequence direction was adjusted, exact barcode matching, two-nucleotide mismatch in primer matching.

Operational taxonomic units (OTUs) with 97% similarity cutoff were clustered using UPARSE version 7.1, and chimeric sequences were identified and removed. The taxonomy of each OTU representative sequence was analyzed by RDP Classifier version 2.2 against the 16S rDNA database (eg. Silva v128) using a confidence threshold of 0.7.

### 4.4. Analyses of SCFAs

The samples used for SCFAs analyses were filtered through a 0.22 µm filter. Filtered supernatant, 10 µL, was injected into the High-Performance Liquid Chromatography System (1260, Agilent, Thuringia, Germany) equipped with a Shodex Rspak KC-811 (8.0 × 300 mm) and an ultraviolet detector (210 nm). Eight SCFAs (lactic acid, formic acid, acetic acid, propionic acid, isobutyric acid, butyric acid, isovaleric acid, and valeric acid) were evaluated. Isocaproic acid was used as an internal standard. The eluent (0.01% *v/v* H_3_PO_4_) was used at a flow rate of 0.8 mL/min.

### 4.5. Histological Analyses

Following anaesthetization, the heart, kidney, and lung were rapidly removed from rats. Tissues were weighed and fixed in 4% aqueous formalin before being snap frozen in liquid nitrogen; then, they were stored at −80 °C. The fixed tissues were embedded in paraffin, sectioned in 3 µm portions with a microtome, and stained with Masson’s trichrome reagent to be able to observe the collagen present in heart tissues. Perivascular and interstitial fibrosis levels were quantified in heart using the Olympus B×41 microscope (400× magnification) (Marques, et al., 2017) [17]. Collagen levels were expressed as a percentage of the area of the region using the Image-Pro Plus 6.0 (Media Cybemetics, INC., Rockville, MD, USA).

### 4.6. RNA Extraction and Cardiac Transcriptome Sequencing

To explore the mechanism of reduction of BP and cardiac fibrosis, the cardiac transcriptome of the Vehicle, HPAO, and Captopril groups (*n* = 4 per group) were examined. Total RNA was extracted using the TRIzol^®^ Reagent according the manufacturer’s instructions (Invitrogen, Waltham, MA, USA); genomic DNA was removed using DNase I (TaKara). The RNA quality was assessed by a 2100 Bioanalyser (Agilent Technologies Inc., Palo Alto, CA, USA) and quantified using the NanoDrop 2000. The high-quality RNA sample (OD260/280 = 1.8–2.2, OD260/230 ≥ 2.0, RIN ≥ 6.5, 28S:18S ≥ 1.0, >10 μg) was used to construct the sequencing library. Total RNA (5 µg) underwent mRNA enrichment using the Poly(A) mRNA magnetic isolation method. The transcriptome library was prepared with the TruSeqTM RNA sample preparation Kit for Illumina (San Diego, CA, USA). Libraries were sized according to cDNA target fragments of 200–300 bp on 2% (*w/v*) low-range ultra agaroses followed by PCR amplified with the Phusion DNA polymerase (NEB) for 15 PCR cycles. After DNA was quantified by the TBS380 fluorimeter (Turner Biosystem, Sunnyvale, CA, USA), the paired-end RNA-seq sequencing library was sequenced with Illumina HiSeq 4000 (2 × 150 bp read length). RNA-seq sequencing was undertaken at the Shanghai Majorbio Bio-Pharm Technology Co. Ltd. (Shanghai, China).

### 4.7. Bioinformatic Analyses

Raw paired end reads were trimmed, and their quality was controlled by SeqPrep and Sickle. Clean reads were separately aligned to the reference genome with the orientation mode using TopHat (version 2.0.0) [56]. R statistical package software EdgeR (Empirical analysis of Digital Gene Expression in R) [57] was utilized for the differential expression analysis. GO functional enrichment and Kyoto Encyclopedia of Genes and Genomes (KEGG) pathway analyses were carried out by Goatools and KOBAS [58]. Gene Set Enrichment Analysis (GSEA) was performed using the pathways of the Natriuretic Peptide A (*Nppa*) gene by interrogating the KEGG databases.

### 4.8. Real-Time Quantitative PCR Analyses

The cardiac genes of *Nppa* were analyzed by real-time quantitative PCR (Applied Biosystems StepOnePlus^TM^, Thermo Fisher Scientific, Waltham, MA, USA). After the rat cardiac tissues were homogenized, the total RNA was extracted as described above and reverse transcribed into cDNA with the AccuRT Genomic DNA Removal Kit (Applied Biological Materials Inc., Vancouver, BC, Canada). Real-time quantitative PCR was performed with the Eva Green 2× qPCR MasterMix (Applied Biological Materials Inc., Vancouver, BC, Canada). The amount of target RNA was normalized to the amount of endogenous GAPDH control, and the results were given by 2^−ΔΔCT^ relative to the control sample. Primers used in the amplification reaction were forward: 5′-CGGAAGCTGTTGCAGCCTA-3′ and reverse: 5′-GCCCTG AGCGAGCAGACCGA-3′.

### 4.9. Statistical Analysis

The GraphPad Prism (version 5) package and origin packages (version 9.0) were used for generating graphs. One-way ANOVA for multiple comparisons was used to compare the Vehicle and HPAO, LPAO, KCl, and Captopril groups. All data are presented as mean ± SD or mean ± SEM; those with a *p* < 0.05 were considered statistically significant.

## 5. Conclusions

This study suggests that PAO could lower BP and improve the development of HF in SHR by modulating GM, such as reducing the ratio of Firmicutes to Bacteroidetes, the relative abundance of *Prevotella*, *Phascolarctobacterium*, and decreasing the plasma levels of LPS metabolites produced by the Gram-negative bacteria. PAO could regulate the expression of *Nppa* gene, ANP, and AngII levels in plasma, thus protecting the cardiac function. PAO has great potential in HTN therapy.

## Data Availability

The data are available from the corresponding author on reasonable request.

## Supplementary Materials

The following are available online at https://www.mdpi.com/article/10.3390/ijms22189823/s1.
