# Supplementary material for: Potassium Alginate Oligosaccharides Alter Gut Microbiota, and Have Potential to Prevent the Development of Hypertension and Heart Failure in Spontaneously Hypertensive Rats
Source: Int J Mol Sci. 2021 Sep 11;22(18):9823. doi: 10.3390/ijms22189823 (PMC8470992; doi:10.3390/ijms22189823)
Supplement: Supplementary file 1 [file ijms-22-09823-s001.zip › ijms-1329937-SI.pdf]

Supplementary materials

Potassium Alginate Oligosaccharides Alter Gut Microbiota, and Have Potential to Prevent the Development of Hypertension and Heart Failure in Spontaneously Hypertensive Rats

Zhen-Lian Han [1,2], Meng Chen [2], Xiao-Dan Fu [2], Min Yang [3], Maria Hrmova [1], Yuan-Hui Zhao [2,*] and Hai-Jin Mou [2,*]

Supplementary Figures

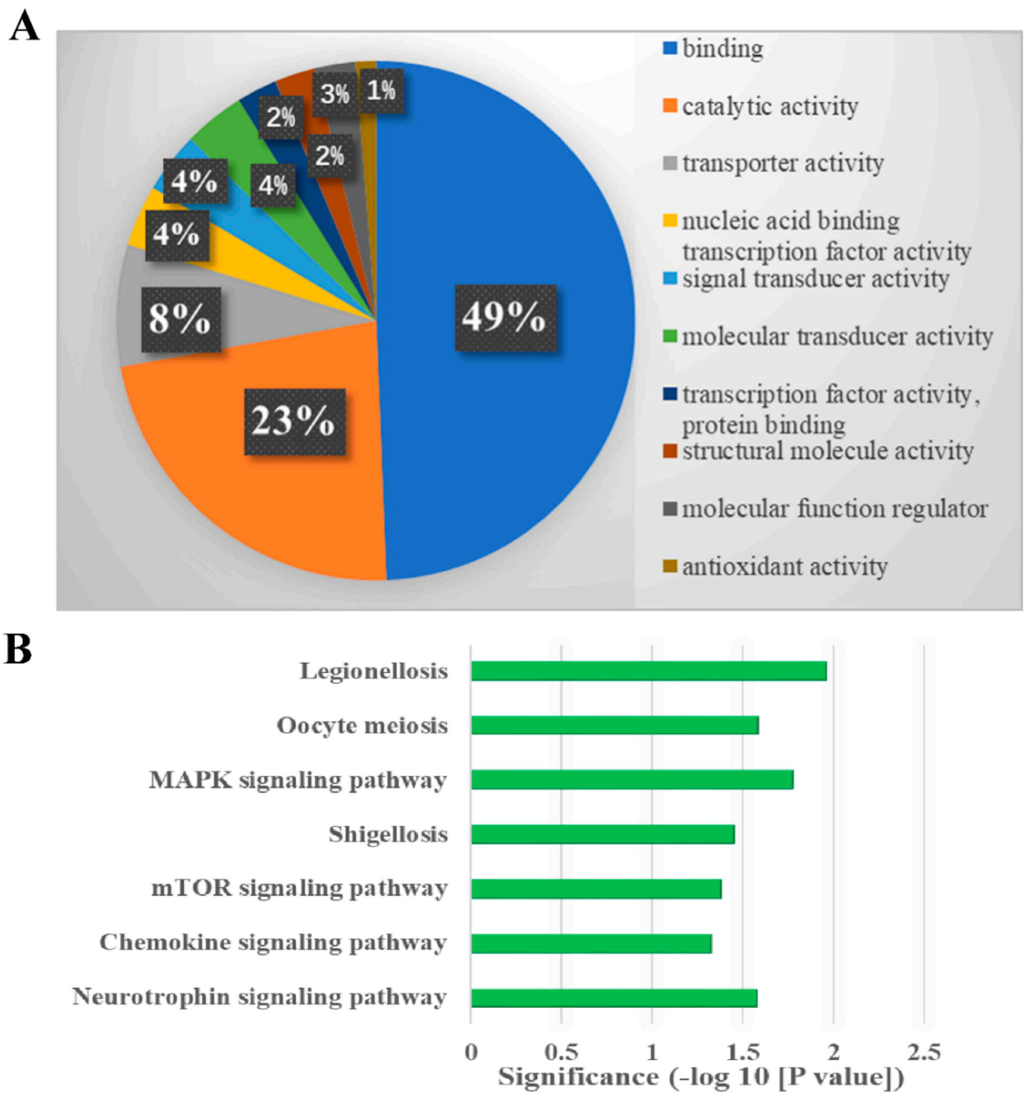

Figure S1. (**A**) Molecular function of DEG entries in percent (Vehicle *vs*. HPAO) examined *via* GO analyses, and (**B**) Seven significantly KEGG pathways of DEG entries in the HPAO and Vehicle groups.

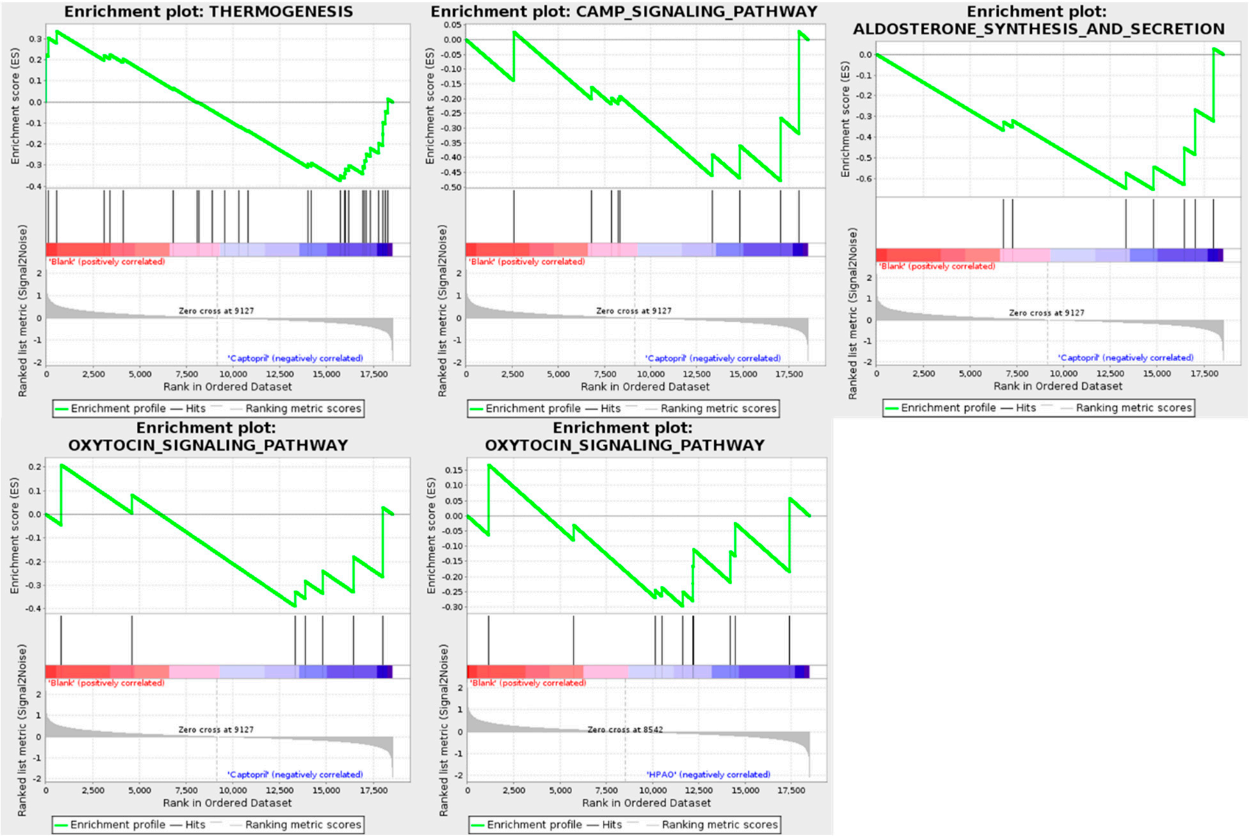

Figure S2. Captopril up-regulates thermogenesis, the cAMP signaling pathway, aldosterone synthesis and secretion, and the oxytocin signaling pathway, while the HPAO supplementation also up-regulates the oxytocin signaling pathway in cardiac tissue.

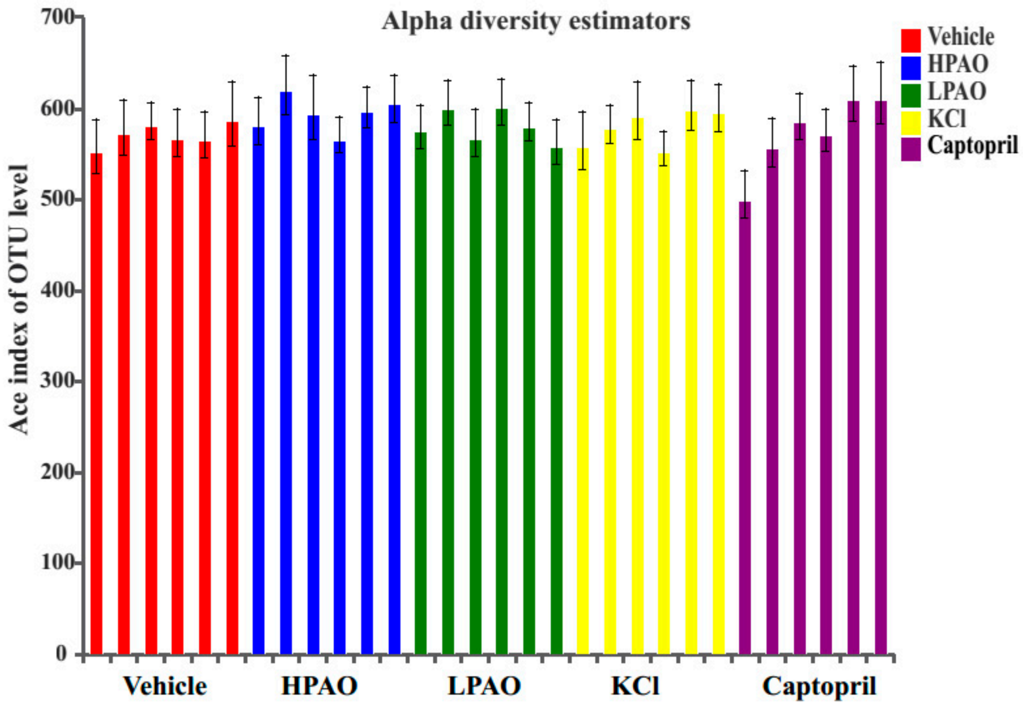

Figure S3. Alpha diversity estimates of ACE in various groups.

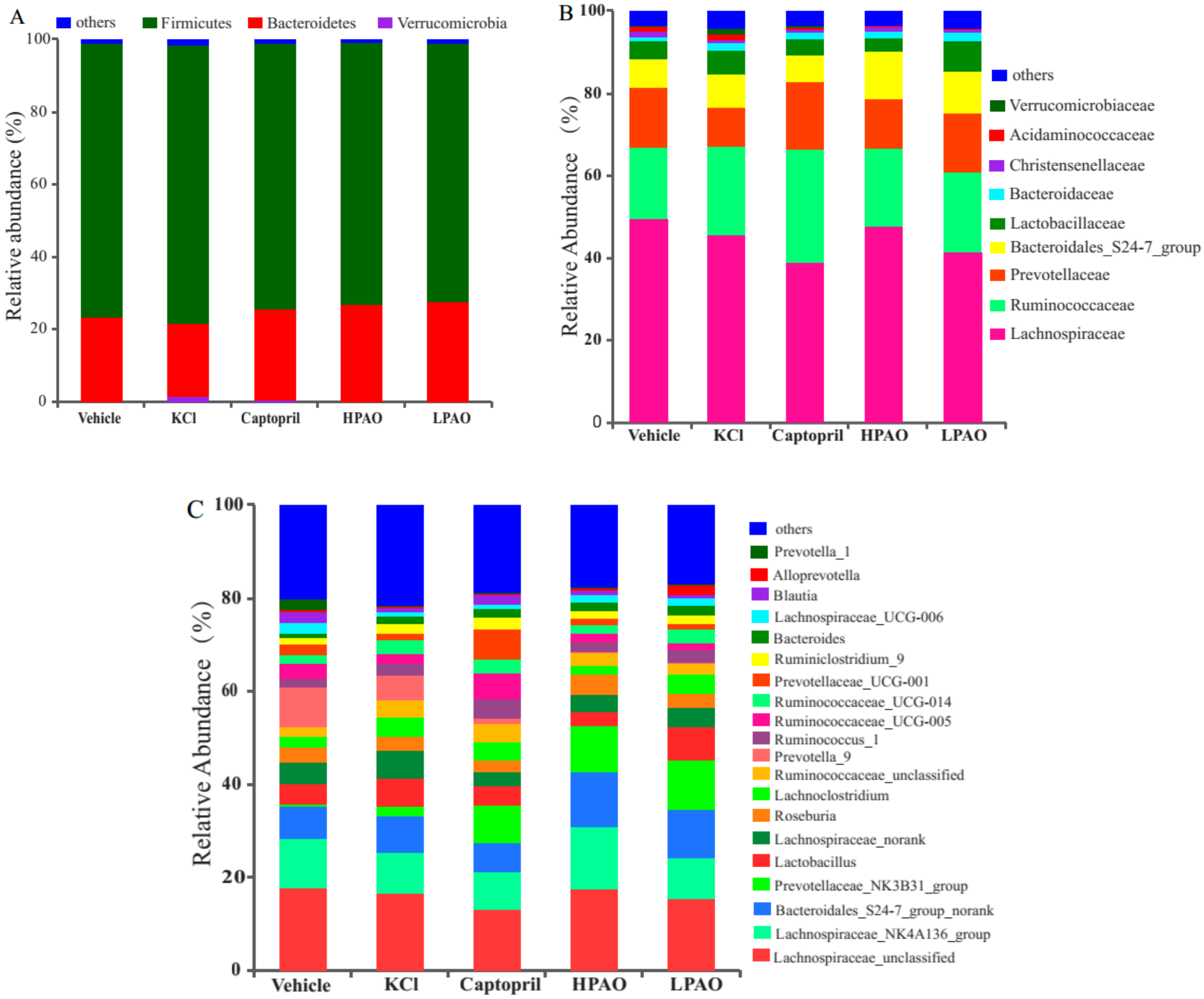

Figure S4. Differences in abundance in bacterial phyla (**A**), families (**B**) and at genera level (**C**) between the diet groups.

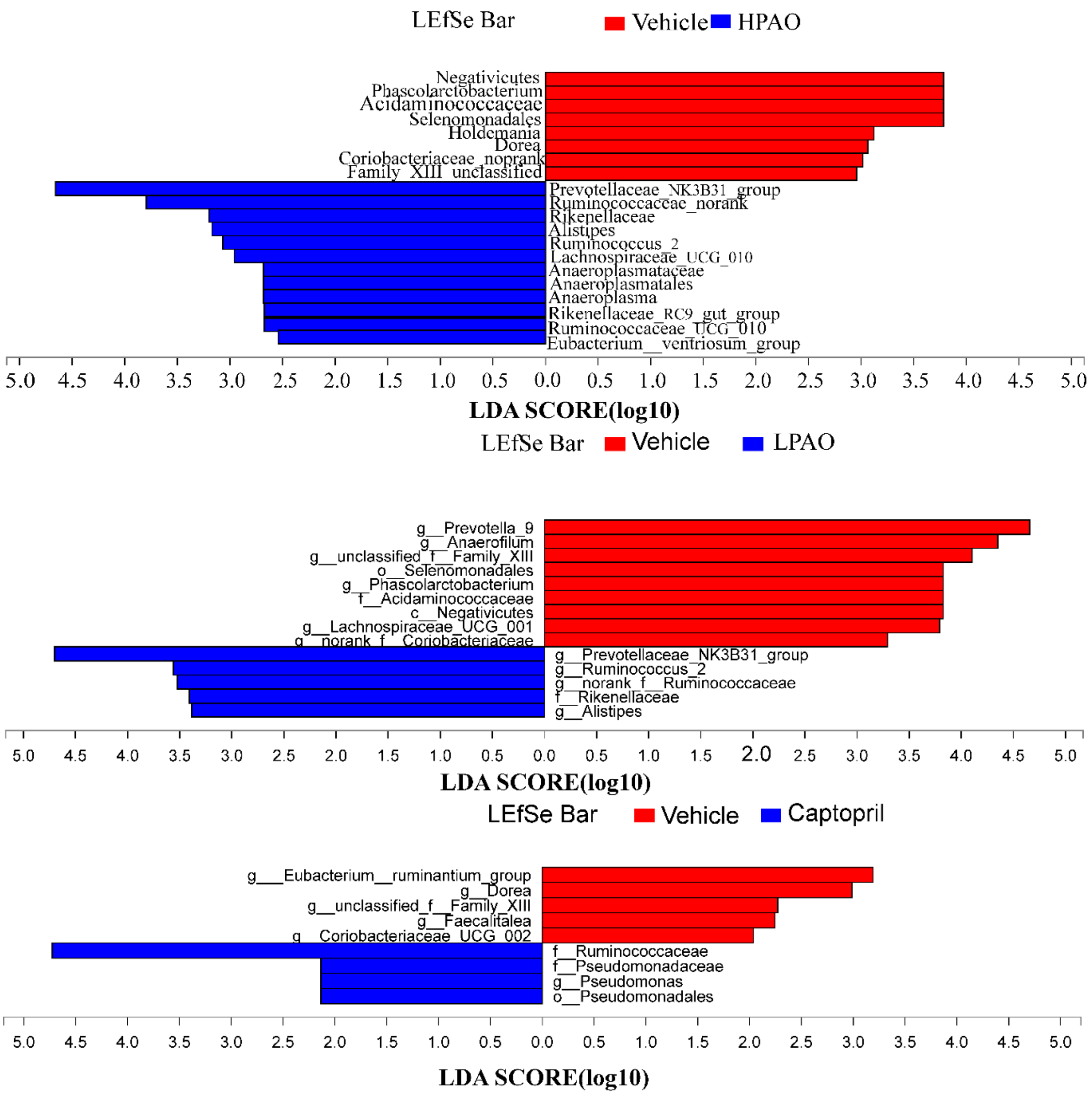

Figure S5. LEfSe analyses of the HPAO, LPAO, Captopril and Vehicle-treated groups.

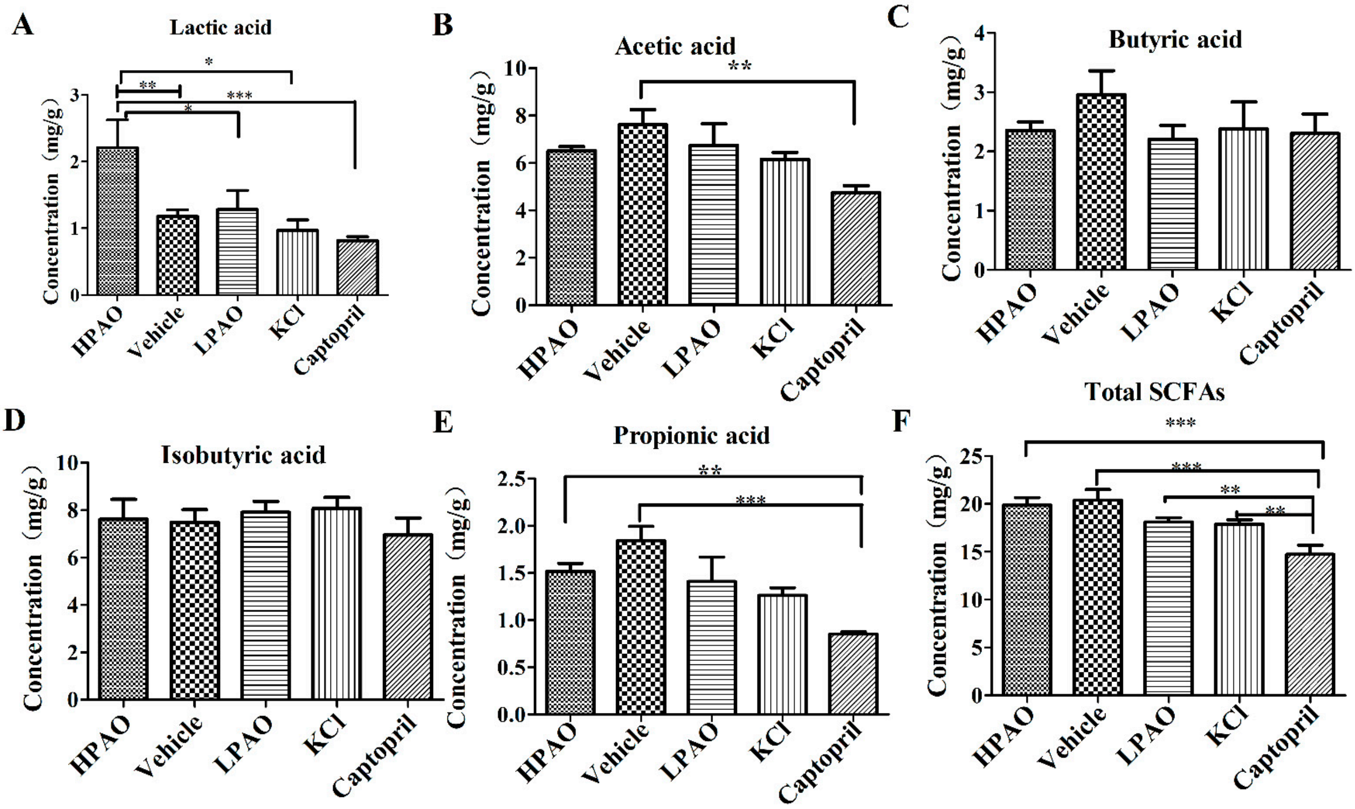

Figure S6. Concentrations of lactic acid (**A**), acetic acid (**B**), isobutyric acid (**C**), butyric acid (**D**), propionic acid (**E**) and total SCFAs (F) (* $p < 0.05$, ** $p < 0.01$, *** $p \leqslant 0.001$).

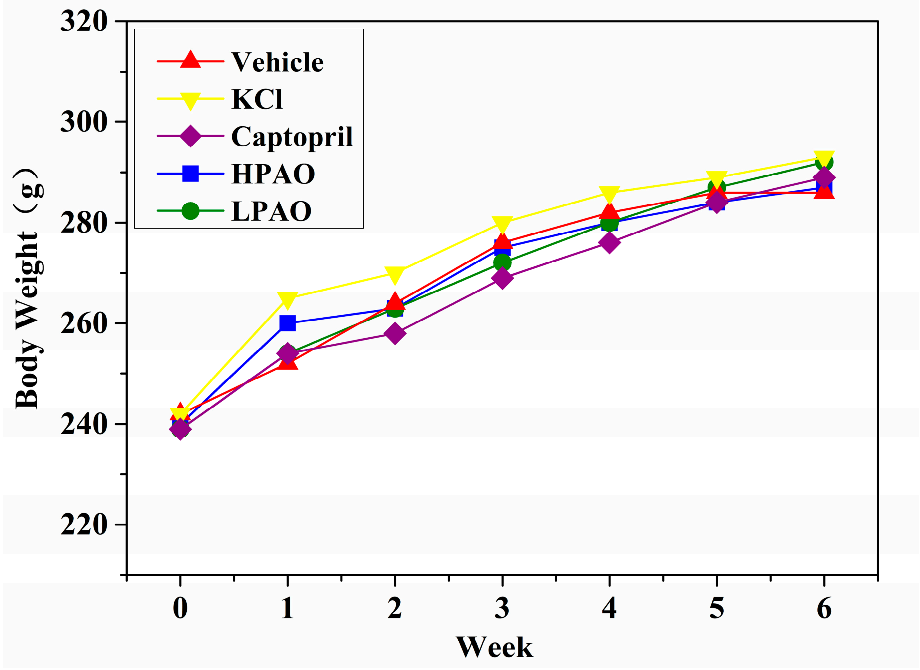

Figure S7. Body weight changes of SHR

Supplementary Tables.

Table S1. Cardiac transcriptome analyses showing DEG entries in SHR fed by HPAO and the Vehicle diets, with FC* > 2 or FC < 0.5.

Gene ID	Gene name	FC	Log2FC	*p* value
ENSRNOG00000011316	*Fam167a*	0.466	-1.10234932	0.000106985
ENSRNOG00000047618	*LOC100910196*	0.337	-1.571132289	0.00039976
ENSRNOG00000057773	*uc_338*	60.734	5.924436185	0.00316099
ENSRNOG00000050419	*Avil*	0.347	-1.527123904	0.003937369
ENSRNOG00000061256	*AABR07006888.1*	6.835	2.773006658	0.004333156
ENSRNOG00000002139	*RGD1311575*	2.711	1.438744565	0.006822292
ENSRNOG00000060228	*AABR07034750.1*	4.373	2.128529191	0.006989063
ENSRNOG00000062197	*Rn60_10_0910.5*	0.194	-2.366651325	0.0073453
ENSRNOG00000025231	*Lrrc29*	6.018	2.589225395	0.007708271
ENSRNOG00000000916	*Katnal1*	7.423	2.892013175	0.007755043
ENSRNOG00000022499	*Sgo1*	0.346	-1.530440305	0.0094363
ENSRNOG00000049829	*AABR07060872.1*	0.484	-1.046406597	0.009643058
ENSRNOG00000052003	*Mkrn2os*	0.424	-1.238431446	0.011232601
ENSRNOG00000051831	*5_8S_rRNA*	0.202	-2.308053361	0.012356985
ENSRNOG00000021663	*RGD1561849*	0.405	-1.302867308	0.012837898
ENSRNOG00000015682	*Kel*	0.385	-1.378242488	0.013576467
ENSRNOG00000034093	*AABR07011951.1*	0.131	-2.935163319	0.013719875
ENSRNOG00000027582	*Dpysl4*	2.807	1.488795294	0.014836969
ENSRNOG00000029996	*LOC103691939*	0.295	-1.76113601	0.015368278
ENSRNOG00000054582	*LOC100134871*	0.475	-1.073003413	0.016608982
ENSRNOG00000059116	*Cux1*	0.099	-3.332206276	0.017389357
ENSRNOG00000004744	*Fam84b*	0.187	-2.415220573	0.018877187
ENSRNOG00000017412	*AABR07001512.1*	0.409	-1.290382681	0.019154272
ENSRNOG00000017681	*Lim2*	0.44	-1.184779711	0.019193731
ENSRNOG00000003160	*RragB*	3.452	1.787360847	0.019651134
ENSRNOG00000000818	*Nrm*	0.468	-1.095562214	0.020146361
ENSRNOG00000060312	*LOC108348197*	0.162	-2.629894342	0.020589227
ENSRNOG00000021851	*Ccl1*	3.381	1.757465095	0.022167829
ENSRNOG00000038784	*Piezo2*	0.371	-1.430865959	0.023051415
ENSRNOG00000058329	*Prrx2*	0.389	-1.362875602	0.023163391
ENSRNOG00000009052	*Igf2bp3*	2.5	1.321915722	0.023236268
ENSRNOG00000060212	*AABR07045416.1*	2.282	1.19056072	0.024305101
ENSRNOG00000003480	*Aim2*	2.039	1.027686544	0.024616156
ENSRNOG00000056269	*AC110709.2*	2.016	1.011456473	0.025539613
ENSRNOG00000053778	*AABR07063248.1*	0.251	-1.993562551	0.026010941
ENSRNOG00000057335	*Clec1b*	0.414	-1.273392954	0.026664588
ENSRNOG00000059997	*Brip1*	0.411	-1.283141799	0.026983701
ENSRNOG00000033056	*LOC691422*	3.287	1.716554548	0.027388432
ENSRNOG00000051253	*LOC102553828*	0.162	-2.622612116	0.027589038
ENSRNOG00000013768	*Defb1*	0.397	-1.332762723	0.028075565
ENSRNOG00000034040	*Rnf113a1*	2.809	1.489907166	0.028780689
ENSRNOG00000055809	*LOC100911769*	0.241	-2.055474672	0.029079533
ENSRNOG00000001193	*Hsf2bp*	0.473	-1.081129052	0.030565504

ENSRNOG00000012878	*Atp7b*	2.103	1.072406066	0.031590581
ENSRNOG00000016046	*Hecw1*	0.342	-1.548912034	0.034664188
ENSRNOG00000008176	*Nppa*	0.491	-1.026425056	0.034789464
ENSRNOG00000046006	*LOC100362783*	0.154	-2.702073529	0.03536434
ENSRNOG00000000850	*Apom*	2.242	1.165066543	0.035441309
ENSRNOG00000055650	*Pou2f2*	2.007	1.005320452	0.035816925
ENSRNOG00000002976	*Maats1*	2.909	1.540466974	0.036005433
ENSRNOG00000038066	*Fam155b*	0.421	-1.246921318	0.03620045
ENSRNOG00000038275	*Adam4*	2.22	1.15063719	0.036695325
ENSRNOG00000031448	*Stpg1*	0.307	-1.70463283	0.038134147
ENSRNOG00000048270	*Knop1*	0.368	-1.440632813	0.038212653
ENSRNOG00000017198	*Hif3a*	2.585	1.370066815	0.038311266
ENSRNOG00000006519	*Tmem107*	0.464	-1.10673162	0.038695444
ENSRNOG00000059443	*Zfp622*	78.692	6.298146677	0.038876629
ENSRNOG00000019719	*Kcna5*	2.058	1.041145314	0.039309178
ENSRNOG00000058083	*Metazoa_SRP*	0.209	-2.255718713	0.039724158
ENSRNOG00000003208	*Ercc6l*	0.403	-1.309898369	0.039769417
ENSRNOG00000050746	*Rn50_16_0006.1*	2.162	1.112518887	0.040259276
ENSRNOG00000050004	*LOC100912306*	0.396	-1.33689234	0.040897698
ENSRNOG00000056849	*Kantr*	2.242	1.164784298	0.041863586
ENSRNOG00000010610	*Hpgd*	0.5	-1.00003936	0.042331243
ENSRNOG00000028581	*Ccdc138*	2.013	1.009457748	0.042904598
ENSRNOG00000062152	*Rn60_Y_0001.1*	2.031	1.022055332	0.044740802
ENSRNOG00000060289	*7SK*	10.764	3.428124009	0.0460798
ENSRNOG00000015168	*LOC365238*	2.504	1.323986284	0.046542167
ENSRNOG00000046379	*LOC100912604*	10.197	3.350084675	0.046589328
ENSRNOG00000025327	*Tert*	2.149	1.103452291	0.046664105
ENSRNOG00000025302	*Cdca2*	0.4	-1.322793051	0.04862952
ENSRNOG00000046560	*AC109096.1*	0.343	-1.544040207	0.048730381
ENSRNOG00000048247	*AABR07028995.1*	22.957	4.520860064	0.049254752
ENSRNOG00000019771	*Tbx6*	2.057	1.040818377	0.049565269
ENSRNOG00000021050	*Olr319*	0.464	-1.109134812	0.049962701

* FC refers to fold change.

Table S2. Cardiac transcriptome analyses showing genes that are differentially expressed in SHR fed by Captopril and Vehicle diet with FC* > 2 or FC < 0.5.

Gene ID	Gene name	FC	Log2FC	*p* value
ENSRNOG00000008176	*Nppa*	0.354	-1.497805226	2.34E-05
ENSRNOG00000031802	*LOC691427*	0.294	-1.767703041	0.000732
ENSRNOG00000010666	*Wisp2*	0.454	-1.138557887	0.00124
ENSRNOG00000018450	*Slc25a22*	2.256	1.173894606	0.002229
ENSRNOG00000009052	*Igf2bp3*	2.983	1.576962574	0.002312
ENSRNOG00000046133	*LOC102553613*	5.49	2.456883223	0.00284
ENSRNOG00000054855	*AABR07014855.1*	2.669	1.416443377	0.003489
ENSRNOG00000054073	*AABR07003650.1*	0.407	-1.296629518	0.00428
ENSRNOG00000012742	*Irx2*	2.138	1.096311559	0.004531
ENSRNOG00000054768	*AABR07050487.1*	8.864	3.148033427	0.004875
ENSRNOG00000003959	*Rgs18*	0.49	-1.030162449	0.004945
ENSRNOG00000046658	*LOC103690116*	117.525	6.876822482	0.005669
ENSRNOG00000048247	*AABR07028995.1*	96.343	6.590101007	0.00833
ENSRNOG00000020025	*Slc29a2*	18.204	4.186162722	0.008645
ENSRNOG00000007943	*Gsdma*	0.374	-1.418764796	0.009141
ENSRNOG00000016983	*Myh7*	0.085	-3.559196042	0.010566
ENSRNOG00000013166	*Wnt4*	3.078	1.621896958	0.012214
ENSRNOG00000055809	*LOC100911769*	0.133	-2.907120204	0.012686
ENSRNOG00000047546	*Gas2l2*	0.034	-4.883204699	0.013544
ENSRNOG00000027433	*LOC100910708*	0.185	-2.434812183	0.013803
ENSRNOG00000015027	*Il18r1*	0.491	-1.024788783	0.013916
ENSRNOG00000061256	*AABR07006888.1*	5.293	2.404161324	0.016385
ENSRNOG00000052264	*AABR07034393.1*	2.302	1.202842864	0.016621
ENSRNOG00000037709	*Armcx1*	0.038	-4.717079635	0.018635
ENSRNOG00000033335	*Cenpi*	2.45	1.292759879	0.018682
ENSRNOG00000051193	*Rn50_2_1408.1*	0.429	-1.220292043	0.018719
ENSRNOG00000033056	*LOC691422*	3.07	1.618274982	0.019928
ENSRNOG00000042496	*Cyp4f5*	0.246	-2.02117437	0.020314
ENSRNOG00000033736	*Diras3*	0.495	-1.014949756	0.020997
ENSRNOG00000017689	*Itih3*	0.341	-1.553054429	0.021655
ENSRNOG00000054955	*Adgra1*	5.004	2.323016825	0.021664
ENSRNOG00000005708	*Mmp16*	0.373	-1.42273851	0.021886
ENSRNOG00000025179	*AABR07045322.1*	5.858	2.550356928	0.022275
ENSRNOG00000001792	*Slc12a8*	0.454	-1.140347384	0.022617
ENSRNOG00000022429	*RGD1312005*	0.281	-1.832674579	0.023259
ENSRNOG00000031448	*Stpg1*	0.325	-1.622324138	0.024792
ENSRNOG00000062197	*Rn60_10_0910.5*	0.328	-1.610324339	0.026229
ENSRNOG00000000007	*Gad1*	2.071	1.05058884	0.026868
ENSRNOG00000057760	*Stil*	0.493	-1.021580059	0.027091
ENSRNOG00000038068	*Pcdh9*	0.356	-1.488702468	0.028528
ENSRNOG00000027582	*Dpysl4*	2.615	1.386803933	0.030477
ENSRNOG00000042596	*Fam205a*	0.475	-1.07456879	0.030594
ENSRNOG00000037607	*LOC100911575*	0.229	-2.124716906	0.030633
ENSRNOG00000000818	*Nrm*	0.464	-1.109081232	0.031667
ENSRNOG00000021945	*Ccdc184*	0.424	-1.238083736	0.031822

ENSRNOG00000025231	*Lrrc29*	5.102	2.351042372	0.031924
ENSRNOG00000062143	*LOC100911830*	2.11	1.077270452	0.032035
ENSRNOG00000059443	*Zfp622*	46.986	5.554145041	0.033804
ENSRNOG00000045973	*LOC102553861*	3.788	1.921516833	0.03529
ENSRNOG00000057773	*uc_338*	39.424	5.300987772	0.035512
ENSRNOG00000019719	*Kcna5*	2.366	1.242629724	0.036733
ENSRNOG00000004498	*Scin*	0.314	-1.673156549	0.036901
ENSRNOG00000058083	*Metazoa_SRP*	0.215	-2.21984493	0.037323
ENSRNOG00000003279	*Syce2*	0.253	-1.984176229	0.039367
ENSRNOG00000009209	*Slitrk1*	0.462	-1.115435354	0.042736
ENSRNOG00000043480	*Timm8a1*	0.481	-1.055288003	0.042917
ENSRNOG00000032224	*LOC100912489*	0.4	-1.323716536	0.043037
ENSRNOG00000062232	*Mcc*	2.178	1.123066533	0.043664
ENSRNOG00000006553	*Bnc2*	0.446	-1.163270038	0.044169
ENSRNOG00000024065	*Zfp575*	0.362	-1.464514665	0.044535
ENSRNOG00000045683	*LOC102553715*	0.383	-1.384118025	0.045676
ENSRNOG00000050786	*AC103024.2*	2.16	1.11090797	0.04616
ENSRNOG00000039571	*Glod5*	2.474	1.306848097	0.047118
ENSRNOG00000050714	*Islr2*	0.445	-1.166829535	0.048297
ENSRNOG00000048545	*LOC100910483*	3.066	1.616145852	0.048359
ENSRNOG00000060289	*7SK*	9.676	3.27447061	0.048704
ENSRNOG00000004795	*Pof1b*	0.44	-1.184838406	0.049054

* FC refers to fold change.

Table S3. Cellular component of DEG entries in percent (Vehicle *vs.* HPAO) evaluated by GO analyses.

GO ID	Description	Term Type	Percent (%)
GO:0005623	cell	cellular_component	64
GO:0044464	cell part	cellular_component	61.33333333
GO:0043226	organelle	cellular_component	49.33333333
GO:0044422	organelle part	cellular_component	32
GO:0016020	membrane	cellular_component	30.66666667
GO:0044425	membrane part	cellular_component	21.33333333
GO:0031974	membrane-enclosed lumen	cellular_component	17.33333333
GO:0032991	macromolecular complex	cellular_component	16
GO:0005576	extracellular region	cellular_component	10.66666667
GO:0044421	extracellular region part	cellular_component	10.66666667
GO:0030054	cell junction	cellular_component	4
GO:0099080	supramolecular complex	cellular_component	2.666666667
GO:0019012	virion	cellular_component	1.333333333
GO:0045202	synapse	cellular_component	1.333333333
GO:0044456	synapse part	cellular_component	1.333333333
GO:0009295	nucleoid	cellular_component	1.333333333
GO:0044423	virion part	cellular_component	1.333333333

Table S4. Biological process of DEG entries in percent (Vehicle *vs*. HPAO) evaluated by GO analyses.

GO ID	Description	Term Type	Percent (%)
GO:0009987	cellular process	biological_process	58.66666667
GO:0044699	single-organism process	biological_process	53.33333333
GO:0065007	biological regulation	biological_process	41.33333333
GO:0050789	regulation of biological process	biological_process	36
GO:0050896	response to stimulus	biological_process	34.66666667
GO:0008152	metabolic process	biological_process	33.33333333
GO:0032501	multicellular organismal process	biological_process	32
GO:0071840	cellular component organization or biogenesis	biological_process	28
GO:0051179	localization	biological_process	22.66666667
GO:0032502	developmental process	biological_process	22.66666667
GO:0023052	signaling	biological_process	22.66666667
GO:0048518	positive regulation of biological process	biological_process	21.33333333
GO:0048519	negative regulation of biological process	biological_process	17.33333333
GO:0051704	multi-organism process	biological_process	12
GO:0022414	reproductive process	biological_process	10.66666667
GO:0000003	reproduction	biological_process	10.66666667
GO:0002376	immune system process	biological_process	8
GO:0040011	locomotion	biological_process	5.333333333
GO:0040007	growth	biological_process	1.333333333
GO:0098754	detoxification	biological_process	1.333333333
GO:0022610	biological adhesion	biological_process	1.333333333
GO:0099531	presynaptic process involved in chemical synaptic transmission	biological_process	1.333333333

Table S5. Significant enrichment of 48 KEGG pathways in DEG entries.

Pathway ID	Description	*p* value_uncorrected	*p* value_corrected
map04722	Neurotrophin signaling pathway	0.026567458	0.318809495
map04062	Chemokine signaling pathway	0.046924662	0.321769109
map04150	mTOR signaling pathway	0.041417156	0.331337245
map05131	Shigellosis	0.035209577	0.338011941
map04931	Insulin resistance	0.196917798	0.36354055
map05143	African trypanosomiasis	0.18377674	0.36755348
map04914	Progesterone-mediated oocyte maturation	0.171921293	0.375101004
map00240	Pyrimidine metabolism	0.195467854	0.375298279
map04714	Thermogenesis	0.182303842	0.380460191
map04915	Estrogen signaling pathway	0.230967496	0.382291028
map05200	Pathways in cancer	0.230292158	0.394786557
map05145	Toxoplasmosis	0.223991068	0.398206343
map04612	Antigen processing and presentation	0.27682303	0.40265168
map04010	MAPK signaling pathway	0.0168818	0.405163202
map04012	ErbB signaling pathway	0.168931494	0.405435585
map01524	Platinum drug resistance	0.168931494	0.405435585
map04910	Insulin signaling pathway	0.255583586	0.408933737
map04140	Autophagy - animal	0.289798596	0.40912743
map04666	Fc gamma R-mediated phagocytosis	0.272885826	0.409328739
map05206	MicroRNAs in cancer	0.266277679	0.412300922
map05100	Bacterial invasion of epithelial cells	0.164427199	0.415395028
map05211	Renal cell carcinoma	0.156867489	0.418313305
map04114	Oocyte meiosis	0.026222131	0.419554089
map04621	NOD-like receptor signaling pathway	0.306330376	0.42011023
map04510	Focal adhesion	0.350190577	0.420228692
map05164	Influenza A	0.341877968	0.420772884
map03013	RNA transport	0.337081801	0.425787537
map03040	Spliceosome	0.328606995	0.426300967
map04141	Protein processing in endoplasmic reticulum	0.320027385	0.426703181
map05162	Measles	0.38467952	0.429409697
map05220	Chronic myeloid leukemia	0.152299938	0.430023354
map04015	Rap1 signaling pathway	0.395777041	0.431756772
map04810	Regulation of actin cytoskeleton	0.3779266	0.431916114
map04060	Cytokine-cytokine receptor interaction	0.369957526	0.433121006
map04014	Ras signaling pathway	0.413131326	0.440673414
map05144	Malaria	0.147708445	0.443125335
map05202	Transcriptional misregulation in cancer	0.432078434	0.450864453
map03440	Homologous recombination	0.07587914	0.455274843
map05163	Human cytomegalovirus infection	0.143092889	0.457897245
map04720	Long-term potentiation	0.140002423	0.480008308
map04623	Cytosolic DNA-sensing pathway	0.11009992	0.480436014
map04144	Endocytosis	0.47593507	0.486061348
map05166	Human T-cell leukemia virus 1 infection	0.489188682	0.489188682
map03460	Fanconi anemia pathway	0.095586754	0.509796019
map04213	Longevity regulating pathway - multiple species	0.138453148	0.511211624
map00983	Drug metabolism - other enzymes	0.108498544	0.520793011
map05134	Legionellosis	0.011018774	0.528901174
map05212	Pancreatic cancer	0.136901172	0.547604689

Table S6. Shanon, Simpson, Ace, Chao and Coverage indices were compared between HPAO, LPAO, KCl, Captopril and the Vehicle-treated groups.

Item	*p* value (Vehicle -HPAO)	*p* value (Vehicle -LPAO)	*p* value (Vehicle -KCl)	*p* value (Vehicle-Captopril)
Shannon	0.3072	0.6382	0.07569	0.7722
Simpson	0.6187	0.974	0.1024	0.9262
Ace	0.02999	0.2997	0.4224	0.9468
Chao	0.4395	0.2632	0.7712	0.7548
Coverage	0.8247	0.216	0.9916	0.3281

Table S7. LEfSe analyses of the HPAO, LPAO, Captopril and Vehicle-treated groups.

Name	Type	Phyla	Enrichment
Acidaminococcaceae	family	Firmicutes	Vehicle
Negativicutes	class	Firmicutes	
Selenomonadales	order	Firmicutes	
Coriobacteriaceae	family	Actinobacteria	
Dorea	genus	Firmicutes	
Prevotellaceae_NK3B31_group	genus	Bacteroidetes	HPAO
Norank_Ruminococcaceae	genus	Firmicutes	
Rikenellaceae	family	Bacteroidetes	
Alistipes	genus	Bacteroidetes	
Ruminococcus_2	genus	Firmicutes	
Lachnospiraceae_UGG_010	genus	Firmicutes	
Anaeroplasmataceae	family	Tenericutes	
Anaeroplasmatales	order	Tenericutes	
Anaeroplasma	genus	Tenericutes	
Rikenellaceae_RC9_gut_group	genus	Bacteroidetes	
Ruminococcaceae_UGG_010	genus	Firmicutes	
Eubacterium_ventriosum_group	genus	Firmicutes	
Prevotellaceae_NK3B31_group	genus	Bacteroidetes	LPAO
Ruminococcus_2	genus	Firmicutes	
Norank_Ruminococcaceae	genus	Firmicutes	
Rikenellaceae	family	Bacteroidetes	
Alistipes	genus	Bacteroidetes	
Ruminococcaceae	family	Firmicutes	Captopril
Pseudomonadaceae	family	Proteobacteria	
Pseudomonas	genus	Proteobacteria	
Pseudomonadales	order	Proteobacteria	

Table S8. Chemical composition of PAO.

Item	Massn (Da)	Potassium content (%)	Sodium content (%)	M/G* ratio
PAO	3,389	19	< 2	2.7

*M/G ratio refers to the ratio of mannuronic acid (M) and guluronic acid (G).
